# Wnt Signalling in Gastrointestinal Epithelial Stem Cells

**DOI:** 10.3390/genes9040178

**Published:** 2018-03-23

**Authors:** Dustin J. Flanagan, Chloe R. Austin, Elizabeth Vincan, Toby J. Phesse

**Affiliations:** 1 Molecular Oncology Laboratory, Victorian Infectious Diseases Reference Laboratory and the Doherty Institute, University of Melbourne, Melbourne, VIC 3000, Australia; dustin.flanagan@unimelb.edu.au; 2 Cancer and Cell Signalling Laboratory, European Cancer Stem Cell Research Institute, School of Biosciences, Cardiff University, Cardiff CF24 4HQ, Wales, UK; austincr@cardiff.ac.uk; 3 School of Pharmacy and Biomedical Sciences, Curtin University, Perth, WA 6102, Australia

**Keywords:** stem cells, Wnt signalling, epithelium, cancer, regeneration, plasticity, intestine, stomach, gastric

## Abstract

Wnt signalling regulates several cellular functions including proliferation, differentiation, apoptosis and migration, and is critical for embryonic development. Stem cells are defined by their ability for self-renewal and the ability to be able to give rise to differentiated progeny. Consequently, they are essential for the homeostasis of many organs including the gastrointestinal tract. This review will describe the huge advances in our understanding of how stem cell functions in the gastrointestinal tract are regulated by Wnt signalling, including how deregulated Wnt signalling can hijack these functions to transform cells and lead to cancer.

## 1. Introduction

### 1.1. Adult Stem Cells

Adult stem cells play a vital role in multicellular organisms by maintaining normal tissue homeostasis by ensuring sufficient cells are produced to populate the tissue [1]. They constitute a small population of cells that are defined as having the capacity of both long-term self-renewal and the ability to give rise to at least one type of differentiated cell [2]. Numerous types of precursor cells have been isolated in adult tissues, leading to the concept that organs contain a population, or several populations, of resident stem cells to maintain tissue homeostasis [3].

Prevailing models assume the existence of a single quiescent population of stem cell residing in a niche, however, recent evidence [4] demonstrates the existence of long-lived yet actively cycling stem cell populations. This suggests that clusters of quiescent and active adult stem cells may cohabit the same tissue so that the demands of tissue maintenance and repair can be shared between populations, rather than heaping all cellular responsibilities on a single population [5]. Recent discoveries in the gastrointestinal (GI) tract suggest a model of co-operative stem cell hierarchy whereby proliferative stem cells meet the day-to-day requirements of the tissue and quiescent ‘reserve’ stem cells facilitate tissue regeneration following cytotoxic damage [6].

Several populations of adult stem cells have been identified as the cell of origin for cancer [7]. Stem cells self-renew and have the capacity to promote regeneration, a process similar to tumourigenesis, and therefore it is not surprising that oncogenes, or loss of tumour suppressor genes, are able to hijack these functions to transform these cells [8,9]. Therefore, identification of these cells is also important to help fully understand cancer biology, as well as normal tissue functions. 

Although the function of stem cells, and how they are regulated is not fully understood, the past 15 years has revealed many of the complex signalling events controlling stem cells and identified a critical interaction between stem cells and the microenvironment, or niche, in which they reside. A stem cell niche is composed of other cell types including epithelial and stromal cells, signalling factors and extracellular matrix, which in combination with stem cells intrinsic characteristics, defines their properties and potential [10].

The GI tract is a challenging environment for its cellular constituents, most notably the epithelium, which is at the frontline of harsh luminal contents and mechanical stress. Fundamental to prolonged survival, the GI epithelium has evolved the capacity to remove any exhausted cells and replenish the epithelium with spritely participants. Importantly, GI epithelial renewal is powered by resident stem cells, which depend on the coordinated effort of various cellular and biochemical components. This review will focus on the remarkable progress made over the past 15 years in which Wnt signalling has emerged as one of the critical signalling pathways regulating GI tract stem cells. Although the oesophagus is also part of the GI tract, the identification and function of stem cells in this organ has yet to be fully established with the same level of detail as in the stomach and intestine and thus is not covered in this review [11,12].

### 1.2. Wnt Signalling

Wnt signalling can regulate several cellular functions, including proliferation, differentiation, migration, apoptosis and migration [13]. As such, it is critical during embryonic development, and regulates homeostasis and stem cell function in several adult tissues including the intestine [14,15,16,17], stomach [18], breast [19] and liver [20] (Table 1). The first *Wnt* gene, *Wnt1* (originally named *Int-1*), was identified in 1982 by Nusse and Varmus who described the cloning of a new murine proto-oncogene [21]. It was then observed that its fruit fly homolog was wingless (*Wg*), a gene essential in segment polarity during embryonic development [22]. Further investigation since has shown that *Wnt1* belongs to a family of highly conserved genes known as the wingless-type mouse mammary tumour virus (MMTV) integration site (Wnt) gene family. There are currently 19 known Wnt genes in the genomes of mice and humans [23], which transmit signals via three different pathways; the canonical Wnt pathway, the non-canonical planar cell polarity pathway, and the non-canonical Wnt/calcium pathway [24]. Although distinct, there is considerable cross-talk between each Wnt pathway, and thus Wnt signalling can be considered more broadly as a signalling network containing distinct arms.

Wnt ligands are secreted glycoproteins that activate signalling pathways through the interaction with a cell surface receptor of the frizzled (FZD) family [39,40]. There are 10 FZD genes in mammals, which can form complexes with other FZD proteins and associate with co-receptors such as low-density lipoprotein-related protein (LRP), most commonly LRP5/6, ROR2 or Ryk to transmit signalling into the cell [41,42]. In the canonical pathways, the absence of Wnt ligands leads to the phosphorylation of β-catenin by the destruction complex, which contains the scaffold protein Axin, APC, GSK3β and CK1-alpha. The phosphorylated β-catenin is targeted for proteasomal degradation. This absence of nuclear β-catenin initiates a complex of TCF/LEF and Groucho to recruit HDACs to repress target genes. Upon binding of Wnt to FZD and LRP co-receptors, the LRP receptors are phosphorylated by CK1-alpha and GSK3β [43] which recruit disheveled (DVL) and axin proteins to the plasma membrane where they become polymerized and activated [44]. The DVL polymers inactivate the destruction complex resulting in the stabilization and accumulation of β-catenin, which translocates to the nucleus [45]. Once in the nucleus, β-catenin forms an active complex with lymphoid enhancer factor (LEF) and T-cell factor (TCF) proteins by disarming TLE/Groucho repression and recruiting histone modifying co-activators, such as Pygo and bcl9 [46] to initiate target gene transcription. 

Wnt signalling that is independent of β-catenin is referred to as the non-canonical pathway where a transcriptional response is elicited via an alternative mode of downstream signalling not involving β-catenin–TCF or β-catenin-LEF [24]. There is currently a myriad of non-canonical Wnt pathways which are classified based on the configuration of FZDs and co-receptors involved as well as their downstream effects, however they can be broadly categorized into two pathways; the planar cell polarity (PCP) pathway and the Wnt/Calcium (Wnt/Ca^2+^) pathway.

The PCP pathway was originally identified in *Drosphila* when phenotypes to hairs, bristles and ommatidia were observed in mutants including *Fzd*, *Dsh*, *Celsr*, *Vangl* and *Prickle*. These genes code for proteins that transmit PCP signalling to regulate cell polarity within the plane of the epithelial sheet making the PCP pathway vital for many developmental processes [47,48]. This pathway is activated when Wnt5a, Wnt7a, Wnt8b or Wnt11 bind to FZD receptors and their respective receptor tyrosine kinases; Ror2 or Ryk [49]. This variety of Wnt ligand, FZD receptors and co-receptors allows for significant variation in the transmission of the signal and depending on which complex is activated determines which cytoplasmic PCP pathway signal transduction components are activated such as c-JUN N-terminal Kinase, DVL, Cdc42, RhoA and Rac1 [50]. PCP signalling is also activated when Fzd7 associates with Syndican4 (Sdc4) and R-Spondin (R-Spo) which transmits Wnt5a signals via the internalization of the whole receptor/ligand complex [51,52,53]. Recent data has indicated that PCP signalling may also promote proliferation in an assortment of different cancers [54,55,56,57,58].

The Wnt/Ca^2+^ pathway was first identified in zebrafish embryos where over-expression of Wnt5a was observed to increase the frequency of Ca^2+^ signalling [59] and activate protein kinase C (PKC) and calcium/calmodulin-dependent protein kinase in Xenopus [60]. Wnt/Ca^2+^ signalling is initiated by the interaction of the Wnt/Fzd receptor complex along with the participating co-receptor Ror1/2 which leads to the production of inositol 1,4,5-triphosphate (IP3) and 1,2-diacylglycerol (DAG) as well as calcium via the action of membrane-bound enzyme phospholipase C (PLC). The PLC modifies IP3 and DAG which allows IP3 to diffuse through the cytosol and interact with the calcium channels on the endoplasmic reticulum which results in the efflux of calcium ions; DAG and the intracellular calcium activate PKC. In conjunction with calmodulin, calcium also activates calmodulin dependent protein kinase II (CaMKII). PKC and CaMKII activate numerous regulatory proteins, such as CREB and NFκB, which regulate target gene expression [61].

The formation of Wnt/Fzd/Lrp6 signalling complexes has traditionally been viewed to transduce a linear signal transduction cascade, which centres around stabilization of β-catenin, colloquially known as canonical Wnt signalling. However, recent elegant work from Christof Niehrs and colleagues has shown this pathway has the capacity to bifurcate at the level of Wnt/Lrp6 to initiate other cellular processes independent of β-catenin [62]. For example, Wnt/Lrp6 signalling can sequester up to 70% of the cellular pool of GSK3 in multivesicular bodies, which prevents the many targets of GSK3-mediated phosphorylation being ubiquitylated and degraded [63]. As such, Wnt-dependent stabilization of proteins (Wnt/STOP) peaks during mitosis to slow protein degradation as cells prepare to divide, which influences not only cell-cycle progression but cytoskeletal dynamics, endolysosomal biogenesis and DNA remodelling [62].

The decision of which pathway will be engaged is dependent on the availability of co-receptors and Wnt ligands, thus providing huge complexity and redundancy in the system. As such, there is considerable overlap between the Wnt pathways, for example, Wnt5a/Ca^2+^ signalling can negatively regulate the canonical Wnt signalling pathway [64,65] however, in other contexts Wnt5a is able to activate β-catenin-dependent transcription in the presence of Fzd4 [66]. In addition, Wnt5a can work in co-operation with the Ror2 receptor to promote β-catenin degradation independently of GSK3 and therefore inhibit Wnt3a-medicated canonical Wnt signalling [67,68].

Despite there being extensive knowledge surrounding canonical Wnt signalling in the GI tract, both in normal homeostasis and cancer, the function of non-canonical Wnt signalling has been less well characterised; this is in part due to the lack of suitable reagents and robust assays. Having said that, Wnt5a, a classical non-canonical Wnt, is critical for mucosal healing and regeneration in the colon following injury. Wnt5a plays an instructive role via potentiating TGFβ signalling to limit the proliferation of intestinal stem cells (ISCs) at the site of injury [31], which highlights the multiplexed control of ISCs by Wnt signalling. Non-canonical Wnt signalling is also involved with migration mediated via Fzd7 [69] as well as being critical for the survival, invasion and metastatic capabilities of colon cancer cells [70]. However, the exact relation of Fzd7 with non-canonical signals in CRC is unknown. Although it is well documented that *Wnt5a* is overexpressed in both CRC [71] and GC [32] the precise mechanisms of the non-canonical pathways involvement are less well understood. It has been shown that compared to normal tissue, *ROR2* is frequently downregulated in gastric carcinoma tissues, which suggests that ROR2 has a tumour suppressive role in gastric carcinoma [72]. The exact underlying mechanisms by which ROR2 acts and how the canonical and non-canonical pathways interact requires further investigation.

## 2. Intestinal Stem Cells and Wnt Signalling

### 2.1. Biology of the Intestinal Epithelium

**Figure 1:**
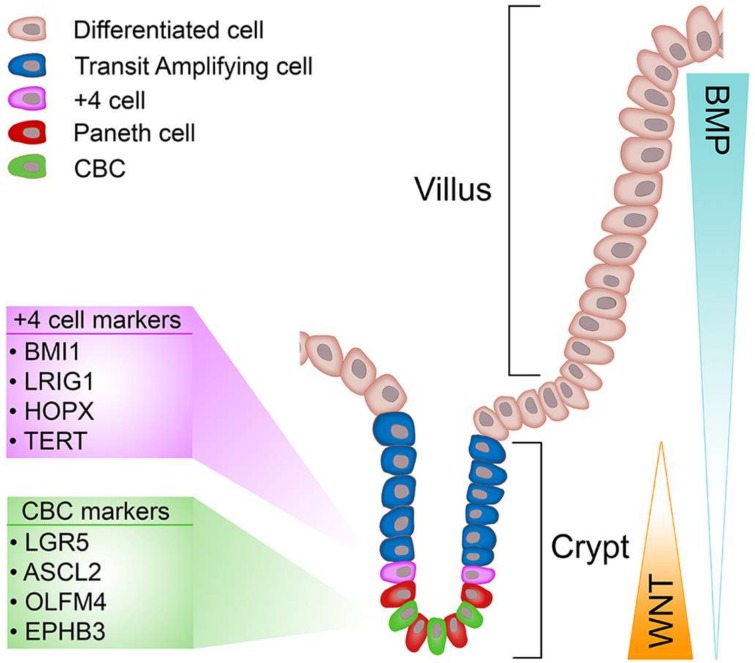
Stem cell populations and signalling gradients of the gastrointestinal epithelium. Schematic of the small intestinal epithelium depicting the various cell types, stem cell populations and signalling gradients.

The epithelial lining of the intestine allows efficient exchange and absorption of nutrients whilst simultaneously excluding passage of harmful molecules and organisms, and undergoing constant renewal [73]. For these reasons the intestinal epithelium represents an excellent model to study the processes that regulate cell renewal, differentiation and homeostasis. The bulk of the simple columnar epithelium of the small intestine is composed of finger-like projections known as villi that extend into the intestinal lumen and house the various cell types needed for nutrient exchange and absorption [74]. At the bases of villi are mucosal invaginations, the crypts of Lieberkühn, (referred from herein as crypts) which are home to progenitor cells, differentiated Paneth cells and ISCs (Figure 1). The colon lacks villi, but still retains the crypt-like structures, which house the stem cells, located in the base, and the differentiated progeny [75]. The colon absorbs water, including water with ions, vitamins and nutrients dissolved in it from host gut bacteria in all the differentiated cells. Variable chemical, biological and mechanical stresses encountered by the intestinal epithelium stimulate a perineal renewal along a vertical (crypt-villus) intestinal axis every four to five days, which serves as a protective mechanism to rid the epithelium of any cells that have undergone genotoxic insult. The driving force behind epithelial renewal of the intestine are ISCs that proliferate daily [4,76] to generate a population of unspecified transit-amplifying (TA) cells that rapidly divide while migrating vertically along an epithelial conveyer belt to produce new secretory, enteroendocrine and absorptive lineages that replenish exhausted cells at the villus tips (or top of the crypt in the colon), which undergo apoptosis and are shed into the lumen. However, secretory Paneth cells in the small intestine, or cKit+/Reg4+ cells in the colon [77,78], do not follow the rapid renewal and migration pattern displayed by other intestinal cell types; Paneth cells are renewed every 3-6 weeks by committed secretory progenitor cells located at the base of the TA compartment, which mature into fully differentiated Paneth cells as they migrate toward the crypt base. Paneth cells play important roles in controlling the ISC microenvironment through secretion of antimicrobial peptides (defensins and lysozyme) and various growth factors that confer ‘stemness’ (Wnt, EGF and Notch) [79,80]. Of these factors, extensive research demonstrates Wnt signalling as a critical regulator of ISC maintenance. However, when Wnt signalling is deregulated it can provide favourable conditions to transform cells [13].

### 2.2. A Brief Perspective of Wnt Signalling in the Intestine

To understand why Wnt signalling plays such an instrumental role in ISC biology, we need to understand how and why Wnt appeared on the radar of gastrointestinal researchers. Near the turn of the 20th century, several groups mapped and functionally linked *APC* (previously discussed), located on chromosome 5q21, to sporadic colorectal cancer (CRC) and familial adenomatous polyposis (FAP), the latter being an autosomal dominant condition that drives the formation of hundreds to thousands of small benign tumours in the large intestine, which can progress to cancer [81,82]. Following these discoveries, immunoprecipitation experiments reveal complexes of APC bound to β-catenin [83], suggesting a role for APC in Wnt signal regulation. Importantly, seminal work published back-to-back in Science show *APC* mutant colorectal cancers fail to regulate β-catenin, which permit constitutive transcriptional activation of a β-catenin/TCF-4 complex, thus driving Wnt signalling in colorectal cancer tissue [84,85]. However, until this point, the requirement of Wnt signalling in the normal intestinal epithelium had not been tested. In the normal intestine, the role of Tcf-4 (encoded by *Tcf7l2*) was elucidated by Korinek et al., which reveal neonatal Tcf7l2−/− mice exhibit a complete absence of the prospective intestinal crypt and stem cell compartments leading to death shortly after birth, highlighting for the first time the necessity of Wnt signalling for intestinal homeostasis and stem cell maintenance [16]. These critical findings were soon reinforced by several key studies that embraced the Cre-LoxP system [86] to permit the investigation of Wnt signalling in the adult intestinal epithelium, which collectively demonstrate that manipulation of key regulators of the Wnt pathway impose an aberrant Wnt-signal gradient, which significantly disrupts intestinal crypt-villus architecture and homeostasis. In addition, FAP patients, in which one allele of *APC* is mutated and Wnt signalling activated when the second is lost via loss-of-heterozygosity, present with polyps in the stomach as well as the intestine, illustrating a role for Wnt in the regulation of gastric stem cells as well [87]. Together these highlight the importance of Wnt signalling for regulating stem cell function in the GI tract, which is the focus of this review [88,89,90,91,92].

### 2.3. Identification and Dynamics of Intestinal Stem Cells

Two prevailing models of ISC identity have competed for legitimacy over the past four decades. The identification of slender, undifferentiated, mitotically active crypt base columnar cells (CBCs) provides the foundation for the “stem cell zone model”. Cheng and Leblond observed the gradual presence of radiolabelled phagosomes, initially detected only in CBCs, in multiple cell lineages. However, the various labelled cell types were observed in separate crypts, negating multipotency of CBCs [93]. Additional evidence in support of CBC stemness came decades later through chemical mutagenesis of the Dlb-1 locus, which generates long-lived clones comprised of all four intestinal cell lineages. This was interpreted as evidence for CBC cells being self-renewing multipotent stem cells [94]. In contrast, studies from Christopher Potten’s group propose cells positioned directly above the uppermost Paneth cell, between +2 and +7, average of +4, relative to the crypt base embody ISCs [76]. Potten’s +4 cells actively divide (daily), but can retain DNA incorporated labels, a trait normally synonymous with cellular quiescence [95]. Moreover, the ability of +4 cells to retain DNA labels while actively proliferating is thought to occur through selective segregation of old (labelled) and new (unlabelled) DNA strands into stem cells and their daughter progeny, respectively, which was proposed as a mechanism to reduce accumulation of DNA damage in long-lived stem cells [96,97]. Furthermore, the radiosensitivity of +4 cells is seen to be a desirable quality for stem cells as it would prevent the accumulation of DNA replication errors that could be inherited by daughter cells. Despite the recent discovery of specific markers for both candidate stem cell populations, demonstrating which of the two models is correct has been immensely complex [73]. However, only recently has a unifying theory that incorporates aspects of both models started to emerge and how each stem cell pool contributes to intestinal homeostasis and regeneration (discussed below).

### 2.4. Markers of CBCs

While it was known that the cellular source of intestinal renewal resided within the crypts, the precise identity of ISCs remained elusive until the beginning of the 21st century. In a landmark study, Barker et al. confirmed a previously identified Wnt target gene, *Lgr5* [92,98], to mark a population of CBCs, which were decades earlier postulated to represent ISCs [93]. Each intestinal crypt harbours approximately 14 Lgr5+ cells, some 10% of which occupy the +4 position, which intercalate between Paneth cells. The generation of a *Lgr5-EGFP-IRES-CreERT2* mouse was used to demonstrate via lineage tracing that mitotically active Lgr5+ cells are self-renewing, long-lived and multipotent [4]. Detailed investigations of the endogenous population dynamics of Lgr5+ cells using the Confetti reporter mouse and mathematical modelling reveal Lgr5+ cells drift towards clonality and divide symmetrically, implying functional equivalence within the Lgr5+ ISC pool [99]. Intra-vital imaging further refines this model, showing ISCs compete for access to essential niche factors (Wnt, EGF, Notch) at the crypt base, resulting in central ISCs having a survival advantage over spatially displaced counterparts (border cells), which will maintain stemness and adopt a progenitor fate respectively [100]. Furthermore, as Lgr5+ cells from the *Lgr5-EGFP-IRES-CreERT2* knock-in mouse express GFP, FACS isolated Lgr5GFP+ cells can generate three-dimensional self-renewing ex vivo organoid cultures, complete with a full repertoire of intestinal lineages in the correct ratios and anatomical position, thus providing additional evidence of *Lgr5* as a marker of ISCs [80,101]. Subsequent investigations have shown *Lgr5* marks stem cell populations in many other tissues such as the hair follicle [102], stomach [103], ovary [104], kidney [105], liver [106], mammary [107], pancreas [108], inner ear [109] and taste buds [110].

The utility of Lgr5 as a marker of ISCs has allowed multiple aspects of ISC biology to be better understood, such as the molecular regulators of stem cell identity. Gene and proteomic signatures of Lgr5+ ISCs [111] identify the transcription factor achaete scute-like 2 (*Ascl2*) as a master regulator of ISCs, which co-operate with Tcf-4 and β-catenin to establish a Wnt-driven stem cell program at the crypt base [111,112]. Like *Lgr5*, *Ascl2* specifically marks CBCs and transgenic overexpression or conditional deletion of *Ascl2* result in crypt hypertrophy and rapid stem cell death respectively [113]. However, in the latter scenario, no gross intestinal phenotype is observed due to ‘escaper’ crypts that can replenish the ISC pool [113]. Intriguingly, although *Ascl2* can increase the number of ISCs and its expression is positively correlated with CRC progression [114], forced expression of *Ascl2* in a mouse model of colon cancer doesn’t accelerate tumourigenesis, which suggests ectopic expression in cells that do not normally express *Ascl2*, rather than overexpression, explain the aetiology of *Ascl2* in CRC [115].

The ephrin family of signalling molecules regulate cell-position cues in a wide variety of tissues, including the intestinal crypt-villus axis. Interactions between Eph receptors and ephrin ligands usually facilitate cell repulsion [116] and within the crypt, Wnt signalling inversely regulates the expression of EphB receptors and ephrinB ligands, creating correct compartmentalisation of undifferentiated and differentiated cells [92]. Indeed, *EphB3* is enriched in Lgr5+ cells and *EphB2* is expressed uniformly throughout the crypt, whereas the *ephrinB1* and *B2* ligands are expressed in the villi. Deletion of *EphB2* and *EphB3* triggers a mislocalisation of Paneth cells combined with an abnormal intermingling of proliferative and differentiated cells [117]. Furthermore, paralleling the results in normal crypts, the graded expression of *EphB2* in CRCs identifies tumour cell populations displaying ISC-like or differentiated-like phenotypes [118].

### 2.5. Markers of +4 Cells

Shortly after the discovery of *Lgr5*, several other markers (*Bmi-1, Lrig1, Hopx, m-Tert*) were reported to preferentially mark a population of quiescent cells located above the uppermost Paneth cell, the +4 position, which do not contribute substantially to epithelial homeostasis, but can support epithelial reconstitution following injury [119,120,121,122].

The polycomb complex protein Bmi-1, which regulates hematopoietic and neural stem cell self-renewal, was observed to mark a radio-resistant +4 cell population [120]. In vivo lineage tracing with *Bmi-IRES-CreERT2* mice demonstrate Bmi-1+ cells are multipotent, but do not contribute to epithelial homeostasis under basal conditions [120,123]. While Bmi-1+ cells do participate in epithelial regeneration in response to injury (discussed later), the validity of Bmi-1 as a specific marker of +4 cells was brought into question. Independent analysis show *Bmi-1* expression overlaps substantially with Lgr5+ cells [111,119,122,124] and lineage tracing can initiate anywhere in the crypt, including Lgr5+ cells, and over time, most of these clonal populations are shed from the epithelium demonstrating they originated in short-lived progenitors/stem cells [111]. However, hierarchical clustering of ISC populations by bulk and single-cell mRNA-seq show enteroendocrine (EE) gene signature enrichment in Bmi-1+ cells, which display clonogenic potential ex vivo, suggesting that stem cell activity resides in a subset of Bmi-1+ cells [125,126]. In addition, Bmi-1+ cells are not sensitive to Wnt signalling, as demonstrated by the failure of crypt expansion and atrophy following R-Spo and Dickkopf-1 (Dkk1) treatment respectively, which indicate a functional distinction between Lgr5+ and Bmi-1+ cells [123].

Increased telomerase expression is a desirable trait for stem cells, which buffers against replication-induced senescence. Indeed, loss of telomerase induces crypt-villus atrophy, suggesting a functional role for telomerase maintenance in ISCs [127,128]. Prompted by this hypothesis, Montgomery et al. report a population of rare quiescent cells located in the lower crypt, around the +4 position, labelled by mouse-telomerase reverse transcriptase (*m-Tert*) [122]. *m-Tert*+ cells are radio-resistant and distinct from Lgr5+ cells. In vivo lineage tracing demonstrates a small percentage of m-Tert+ cells are actively cycling stem cells that contribute to intestinal homeostasis [122]. Second-generation Tert knock-in reporter animals (*TertTCE/+*) confirm previous reports of label-retaining qualities displayed by Tert+ cells, but also reveal Tert expression overlaps with ChgA+ cells, which is consistent with multi-capable EE lineages [27,125]. Furthermore, injury-induced activation of Tert+ cells requires Wnt2b signalling, highlighting Wnt as a critical component of successful epithelial recovery following injury [27]. Collectively, these results support *m-Tert* as being a marker of an independent, quiescent damage-inducible population of ISCs [73].

A prominent mitogen that regulates ISCs is Epidermal Growth Factor (EGF) signalling [101]. As such, EGF must be diligently regulated to avoid excessive signalling, which is linked to many epithelial malignancies [129,130]. The leucine-rich repeats and immunoglobulin-like domain 1 (*Lrig1*) gene encodes a single-pass transmembrane receptor that functions as an inhibitor of ErbB signalling in several tissues and marks interfollicular epidermal stem cells [131]. Lineage tracing with *Lrig1-IRES-CreERT2* mice, initiated at +4 position, produce long-lived multipotent clonal units throughout the small intestine [119]. Indeed, models of intestinal injury mobilise Lrig1+ cells to proliferate and regenerate the colonic epithelium [119]. Furthermore, comparative expression analysis of Lrig1+ and Lgr5+ cells reveal different transcriptome profiles, supporting their independence as makers of intestinal (small & large) stem cells [119]. However, an independent study reports contrasting data, bringing into question the specificity of *Lrig1* as a marker of ISCs [132]. In-situ and immunohistochemical analyses confirmed the expression of *Lrig1* in the crypts, however, transcriptome analysis reveal an enrichment of *Lrig1* expression in Lgr5+ cells [132], which is consistent with fluorescent in situ hybridisation (FISH) showing *Lrig1* expression throughout the intestinal crypt [111]. Together these studies highlight an important role for Lrig1 in regulating ErbB signalling in the intestinal crypt, however the broad expression of *Lrig1* and enrichment in Lgr5+ cells suggest it is not a specific marker of +4 ISCs.

The *Hopx* gene codes for an atypical homeobox protein, which is expressed mainly in the +4 cells of the intestine [121]. Hopx+ cells are quiescent, radio-resistant and can restore the epithelium following radiation-induced damage. Unlike Bmi-1+ cells, in vivo lineage tracing from *Hopx-IRES-CreERT2* mice produce multipotent, long-lived clonal tracings throughout the intestinal epithelium [121]. Furthermore, expression profiling of Hopx+ cells and immediate progeny revealed Hopx-derived cells express high levels of *Lgr5* [121]. Conversely, ex vivo organoid cultures derived from isolated Lgr5+ intestinal stem cells can give rise to Hopx+ cells. Taken together, these observations support a model in which actively proliferating Lgr5+ cells and quiescent Hopx+ cells occupy separate positions within the intestinal crypt and can interconvert to maintain epithelial homeostasis and regeneration. 

Overall, the discrepancies between Potten’s original observations of +4 cells and more recent reports highlight differences in radiosensitivity and mitotic activity, which make it difficult to distinguish whether markers of +4 cells truly represent a distinct class of stem cell or whether they are simply progeny of Lgr5+ CBCs that inherit and express different molecular features as they migrate away from the crypt base. However, the gastric epithelium also has distinct populations of stem cells, with several genes identified as markers of reserve stem cells, suggesting that redundancy in stem cells could be an evolutionary conserved function in the GI tract to help it cope with the harsh conditions encountered in this tissue.

### 2.6. Intestinal Plasticity

Following the identification of various ISC markers, questions about ISC hierarchy have been intensely studied. For instance, are ISCs functionally equivalent and are they required during homeostasis and/or regeneration? Given the remarkable capacity of the intestine to regenerate [133,134], models of intestinal regeneration have been utilised to tease-apart the functional requirement of various ISC populations. Calvin Kuo’s group took advantage of differences in sensitivity to ionising radiation to show Bmi-1+ cells are mobilised through epigenetic remodelling to restock the depleted Lgr5+ population and restore the epithelium [123,125]. Similarly, Hopx+ cells can restore the intestinal epithelium following damage, supporting the notion of a dedicated reserve stem cell pool [106]. However, using a diphtheria-toxin (DT) inducible transgenic mouse (*Lgr5-EGFP-DTR*) to selectively ablate Lgr5+ cells highlights the requirement of Lgr5+ cells during intestinal regeneration [135]. Under these conditions (DT + 10γ radiation), the regenerative capability of the intestinal epithelium is significantly impaired, demonstrating that Bmi-1+ and/or Hopx+ cells are unable to mediate efficient intestinal regeneration [135]. Of note, Lgr5 expression is detected in a modest proportion of +4 cells [124], which would also be targeted in experiments using the *Lgr5-EGFP-DTR* mouse. Interestingly, using the same mouse model to selectively ablate the Lgr5+ population under basal conditions reveal no gross alterations to epithelial homeostasis, which is compensated by the activation of Bmi-1+ cells [136]. Importantly, the rapid replacement of Lgr5+ ISCs following ablation has been reconciled by the inherent plasticity of lineage-specific precursor populations. For instance, label-retaining secretory precursor cells, which express a range of ISC markers (*Lrig1, Bmi-1, Lgr5*), under basal conditions give rise to short-lived clones comprised of cells of the secretory lineage [137,138]. Much like the +4 ‘reserve’ population, following injury, secretory precursor cells can generate all epithelial lineages, demonstrating they can de-differentiate into functional multipotent stem cells that subsequently reconstitute the intestinal epithelium [137,138]. Similarly, cells expressing the enterocyte differentiation marker alkaline-phosphatase intestinal (*Alpi*) can be induced to de-differentiate and replenish the Lgr5+ ISC pool following injury [139]. Collectively, these studies suggest a model where actively cycling Lgr5+ CBCs are responsible for the daily turn-over of the intestinal epithelium, whereas a quiescent subset of CBC progeny can restock the active stem cell pool and intestinal epithelium following cytotoxic insult [6]. More importantly, these studies highlight that ISC identity is not an intrinsic quality, but rather defined by a cell’s location within the crypt and exposure to extrinsic ISC promoting factors, including Wnt signals. This current model reconciles the once irreconcilable stem cell zone and +4 models. 

### 2.7. Wnt Signalling Is Critical for ISCs

Wnt signalling plays a critical role in the mammalian intestinal epithelium where it regulates stem cell behaviour, proliferation, cellular differentiation, apoptosis and migration (Table 1). Indeed, much of our understanding concerning the role of Wnt signalling in homeostasis, stem cell biology and disease have originated from studies in the intestinal epithelium as it is a highly dynamic and well characterised system amenable to vast experimentation.

As mentioned earlier, the prominent phenotype observed in Tcf-4 mutant animals highlight a critical role for Wnt signalling in the intestine. Indeed, *Tcf-4* knockdown downregulates key Wnt target genes involved in proliferation and cell-cycle progression such as *Myc*, which normally represses the action of cell-cycle inhibitors such as p21, permitting cell-cycle arrest [92]. Conditional deletion of *Myc* from the intestinal epithelium causes reduced biosynthetic activity and crypt atrophy, which is countered by rapidly clearing Myc-deficient cells from the epithelium, highlighting a critical role for Wnt signalling in maintaining ISC proliferation [140]. Similarly, over-expression of *Dkk1* suppresses Wnt/β-catenin target gene expression resulting in severe lack of proliferative crypts, reminiscent of the phenotype observed in *Tcf-4* mutant mice [16,89,90]. Conversely, hyperactivation of Wnt signalling is sufficient to impose a crypt-progenitor phenotype characterised by mis-localisation of differentiated cell types, impaired differentiation and crypt hyperplasia. Such phenotypes are typically achieved by establishing a situation where β-catenin is unable to be regulated by the destruction complex or by administering the potent Wnt agonist R-Spo [17,141]. However, the latter context is sufficient to bolster the resilience of ISCs following chemoradiotherapy, thereby improving recovery while targeting tumour tissue [142]. Furthermore, conditional deletion of *CTNNB1* (encodes for β-catenin) from the intestinal epithelium disrupts secretory cell specification and triggers rapid repopulation of the epithelium with β-catenin-proficient cells [88]. Studies investigating the mechanisms of how Wnt/β-catenin signalling activates key target genes in the intestine have identified other components of the Tcf/β-catenin transcription complex that play key roles in intestinal homeostasis. Both Brahma-related gene-1 (Brg1) and leukaemia-associated Mllt10/Af10-Dot1l are essential Tcf/β-catenin co-activators required for target gene transcription [143,144]. Targeted deletion of *Brig1* or *Mllt10/Af10-Dot1l* from ISCs results in rapid epithelial repopulation and proliferative defects observed in intestinal crypts respectively [143,144]. 

Competition for niche space is a defining feature in determining ISC identity. Wnt is a central component of the ISC niche, which is provided by neighbouring Paneth cells [80], yet, ablation of Paneth cells reveals no obvious changes to ISCs or intestinal homeostasis [145]. This may have been due to incomplete deletion of all Paneth cells, but nonetheless, conditional deletion of *Wnt3*, the Wnt released by Paneth cells that is required for stem cell function [80], or inhibition of Wnt secretion (conditional deletion of *Porcupine* or *Wntless*) from the epithelium also reveal no change to ISC homeostasis. These data suggest that the surrounding stroma can provide an alternate source of Wnt ligands, and it was recently demonstrated that Wnt2b is secreted by sub-epithelial mesenchymal cells to activate ISCs in situations of Paneth cell shortage [15,28,146]. As mentioned, Wnt is paramount to ISC maintenance, which is reflected by the necessity of Wnt and R-Spo in establishing Lgr5+-derived organoid cultures [101]. Indeed, mesenchymal cells can rescue Wnt-depleted organoid cultures through supplying ISCs with critical Wnts and R-Spo ligands [15]. From the perspective of the ISC, Wnt ligands expressed by Paneth cells (Wnt3a, 6 and 9b) are directly sequestered via binding to Fzd receptors expressed on the surface of ISCs, rather than via intercellular diffusion [33,80,91,147]. We have shown *Fzd7* to be the major Fzd expressed by *Lgr5*+ ISC and deletion of *Fzd7* is sufficient to induce transient depletion of the ISC pool [14]. In addition to its function as a marker of ISCs, *Lgr5* encodes for a 7-transmembrane protein that participates in the Wnt receptor complex where it binds R-Spo [148,149,150] to prohibit the ubiquitylation of Fzd receptors by E3 ligases Znrf3/Rnf43, thereby stabilising Fzd expression, and thus potentiating Wnt signal strength [151,152]. Hence, the R-Spo/Lgr5/Znrf3/Rnf43 signalling axis fine-tunes the level of Wnt at the crypt base, ensuring highest levels are achieved in cells destined for an ISC fate. Indeed, perturbations to any of these Wnt signal regulators induces dramatic changes to the dynamics of the ISC pool and ultimately intestinal homeostasis [141,148,151,152,153]. Recent work has elucidated the roles of Wnt and R-Spo to suggest that R-Spo ligands adopt the primary instructive role in ISC maintenance and proliferation, whilst Wnt ligands confer basal proliferative competence to ISCs through maintaining suitable levels of the R-Spo receptor Lgr5. Thus, in this model, intestinal homeostasis is dictated by the abundance of R-Spo ligand, with Wnt serving to prime the cells which will respond to this signal by upregulating Lgr5 [154]. 

### 2.8. Aberrant Wnt Signalling Triggers Intestinal Tumourigenesis

As mentioned previously, deregulated Wnt signalling is linked to the progression of cancer, most notably CRC [155] (Table 1). Of the three broad phases that encompass CRC tumour evolution, the initiating or ‘breakthrough’ phase is often triggered by truncating mutations to *APC*, providing cells with a growth advantage that progress to small adenomas in the colon [156]. Subsequent mutation to key driver-genes (*Kras*, *Smad4*, *p53*) permit cells to invade and grow in otherwise hostile environments, which over many years progress to adenocarcinoma [157]. Hyperactivated Wnt signalling in CRC can also arise from mutations to *AXIN2* [158] or *CTNNB1* (β-catenin) [85], but account for a small percentage of cases in comparison to *APC*. While the kinetics by which *APC* is mutated differs between FAP and sporadic CRC patients, tumour cells with truncating *APC* mutations that maintain a ‘just-right’ level of Wnt signalling are positively selected for optimal tumour growth [159]. Mouse models of both FAP and sporadic CRC show substantial variation in disease onset and severity based on the nature of *Apc* mutation [160,161], which supports the idea of a ‘just-right’ or ‘Goldilocks’ amount of Wnt signalling as a driver of tumour formation, rather than an “on-off” switch of excessive Wnt activation. Conditional truncation of *Apc* in the intestinal epithelium instantly transforms the epithelium, causing an expansion of ISCs and disruption to epithelial homeostasis [17]. The growth of *Apc*-mutant cells requires the Wnt target *Myc*, which upon co-deletion rescues the intestinal phenotypes induced by *Apc* mutation [162]. Furthermore, restoration of wild-type *Apc* alone is sufficient to block intestinal tumour growth, despite concurrent mutations in oncogenes *Kras*, *Smad4* and the tumour suppressor *p53*, which highlight the clinical importance of controlling Wnt in CRC [163].

Although these early studies provide invaluable insight into the consequences following *Apc* mutation in the intestine, cancer genome sequencing has revealed new kinds of CRC mutations that deregulate Wnt signalling [164,165]. Whole-exome sequencing of patient tumour-matched samples reveal loss-of-function (LOF) mutations to *RNF43*, which negatively regulate Fzd receptors, in 18.9% of CRCs, showing a higher prevalence in microsatellite instable (MSI) tumours [164]. Moreover, *RNF43* mutations are mutually exclusive to *APC* mutations [164]. Conditional deletion of both *Znrf3* and *Rnf43* from the intestinal epithelium induces rapid expansion of Lgr5+ ISCs and subsequent hyperactivation of Wnt signalling, quickly leading to adenoma formation [151]. Similarly, recurrent gene fusions between *R-SPO2* and *R-SPO3* are observed in 10% of CRCs [165], which like *RNF43*, are mutually exclusive to *APC* mutations, indicating a role for R-SPO in Wnt activation in CRC formation. As such, human CRC xenografts treated with a R-SPO3 blocking antibody promotes cell differentiation and decreased Lgr5+ ISC function. Interestingly, a modest reduction in the expression of several Wnt target genes, *AXIN2*, *MYC* and *CCND1*, is observed following anti-R-SPO3 treatment, suggesting therapies targeting R-SPO in the intestine will be well tolerated [166].

Considering the remarkable plasticity of the intestine and long-standing cancer-stem-cell (CSC) theories [7], a question that persists in the field is whether all epithelial cells are equivalent in their tumour forming capability, or are stem cells the only cell type endowed with the potential to give rise to a tumour? Support for the latter hypothesis was shown following targeted activation of Wnt signalling in Lgr5+ or Bmi-1+ ISCs, which lead to rapid adenoma formation [8,120]. In contrast, activation of Wnt signalling in enterocytes failed to yield established adenomas [8]. More recently, two independent approaches have shown LGR5+ cell ablation in human and mouse CRC organoid xenografts is sufficient to induce tumour remission, further supporting a ‘bottom-up’ CSC model for CRC [167]. However, following the cessation of LGR5+ cell ablation, mobilisation of LGR5- cells quickly reinstated tumour growth [168,169], which may undermine the clinical translation of CSC-targeted therapies. Indeed, plasticity within CRC has been previously reported, owing to extrinsic cues from the tumour microenvironment that induce transformation of differentiated epithelial cells through Wnt activation [170]. Similarly, terminally differentiated enterocytes or DCLK1+ tuft-cells re-express ISC markers (*Lgr5, Ascl2, Rnf43*) and acquire tumour-forming capacity following simultaneous stimulation of Wnt and inflammation pathways, which support a ‘top-down’ model of CRC initiation [171,172]. Likewise, TGFβ signalling from the stroma limits the capacity of terminally differentiated enterocytes to de-differentiate following Wnt and MAPK activation, stressing the importance of targeting both cell intrinsic and extrinsic cues in CRC cells [173]. Moreover, elegant work from Simon Leedham’s group show genetic disruption of intestinal morphogen gradients via *GREM1* overexpression enables the persistence of Lgr5- cells to form ectopic crypts on the flanks of villi. This demonstrates selective pressures that disturb homeostatic balance, such as morphogen dysregulation, can cause committed progenitor cells and even some differentiated cells to regain stem cell properties [174]. Collectively, these studies depict a challenging obstacle to successfully treat CRC given the plasticity of differentiated cells to acquire stem cell properties. Thus, it may be an attractive therapeutic approach to target the CSCs and also the niche that provides these signalling cues, including the Wnt pathway.

## 3. Gastric Stem Cells and Wnt Signalling

### 3.1. Gastric Biology

Due to the dynamic function of the stomach, the gastric epithelium is replenished continually, fuelled by a small population of resident adult stem cells [175]. Like the intestine, the gastric epithelium is organized into numerous glandular mucosal invaginations referred to as gastric units, which house stem cells and various differentiated cells responsible for the regular function of the stomach. Gastric units comprise of the flask-shaped gland, which can be subdivided into the isthmus, neck, base and the pit which is continuous with the surface epithelium (Figure 1B). The four terminally differentiated cell types include parietal cells which secrete hydrochloric acid and a number of other factors including HB-EGF, TGF-α and Shh [176], chief cells with pepsinogen granules secreting active pepsin, two types of gastric mucus cells (surface mucous cells and mucous neck cells) secreting Muc5AC and Trefoil Factor-1 (Tff1) or Muc6 and Tff2 respectively [177] as well as several types of hormone-secreting enteroendocrine cells [103]. These lineages differentiate from progenitor cell populations located in the upper neck region of the gastric glands [178]. 

**Figure 2:**
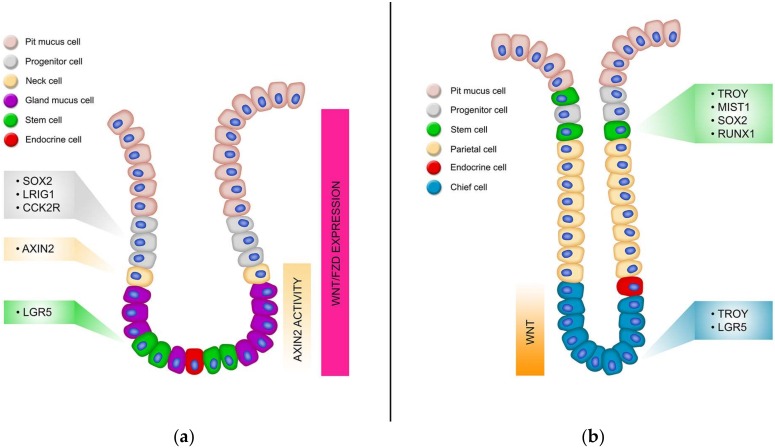
Stem cell populations and signalling gradients of the gastric epithelium. Schematic of the gastric epithelium depicting the various cell types, stem cell populations and signalling gradients. The antral (**a**) and corpal (**b**) epithelium are shown on the left and right respectively.

The glandular stomach is comprised of the proximal corpus and the distal antrum with the precise cellular composition, architecture and turnover rate varying significantly between the two (Figure 2). The interface between the corpus and the antrum is not abrupt but marked by transitional mucosae that shares features of both [179]. The gastric units of the antrum comprise several short glands that feed into a single extended pit. They have a simple cellular composition characterized by abundant gastric alkaline mucous-secreting cells, endocrine cells (G-cells), and low numbers of acid-secreting parietal cells [180,181]. The hydrochloric–secreting parietal cells aide in digestion of food and maintain the acidic pH of the stomach. Large quantities of mucous produced by the mucous-secreting cells protect the duodenum from hydrochloric acid following the emptying of the stomach while the G-cells secrete a variety of hormones that regulate digestion and absorption, such as Ghrelin and Somatostatin [182]. 

In contrast, the corpus gastric units are comprised of several longer tubular glands that feed into short pits. The corpus epithelium is more heterogeneous than that of the antrum, containing many more parietal cells in addition to chief cells, base and neck located mucus cells, and endererndrone cells [175]. Mucous neck cells function in a secretory fashion as well as an intermediate progenitor for chief cells. The chief cells work in conjunction with the HCl-secreting parietal cells and release chymosin and pepsinogen, which is converted to pepsin in acidic conditions thus reflecting its role as the major digestive unit of the stomach [160].

The mechanisms and factors that regulate the homeostasis of the gastric epithelium have only been partially characterized. However, aberrations in these mechanisms, most frequently due to chronic inflammation, can lead to oxyntic atrophy (loss parietal cells) and give rise to Spasmolytic Polypeptide-expressing Metaplasia (SPEM); an event linked to the initiation of both dysplasia and neoplasia [183]. SPEM was first studied in models of acute parietal cell loss using compounds (DMP-777 and L635) to induce parietal cell death via targeted inhibition of protoniophores [184], with similar parietal cell toxicity effects observed following high-dose tamoxifen administration [185]. Although the current understanding is that gastric metaplasia is triggered by the loss of the parietal cells, it has been noted that loss of chief cells also occurs during oxyntic atrophy and recent data postulates that the two processes may be linked. Lineage tracing from chief cells, using the *Mist1-CreERT2:LSL-LacZ* mice identified that SPEM lineages were mainly derived from chief cells which undergo zymogen granule atrophy and changes in gene expression, including upregulation of *Tff2* [186]. Thus, SPEM is a feature that develops in response to gastric mucosal injury, suggesting SPEM aids in regeneration, and in common to the intestine, this is a process that can be hi-jacked by oncogenes or loss of tumour suppressors to initiate tumourigenesis, and consequently SPEM is linked with an increased risk of gastric neoplasia [184,187]. 

### 3.2. Gastric Stem Cells

Gastric epithelial cells are exposed to considerable chemical stress, including low pH and exogenous toxins, and physical stresses from peristaltic contractions of the stomach. To function in this situation, the epithelium has a rapid turnover of cells fuelled by resident populations of stem cells. The corpus and antrum gland are functionally and morphologically distinct and therefore have very different stem cell populations, and turnover rates, with the corpus taking 10–30 days and the antrum much faster at around 7–10 days [175]. Early studies used radioactive nucleotides to label differentiated progenitor populations to investigate gastric epithelial proliferation dynamics [188]. However, the subsequent generation of transgenic mice to mark the progeny of specific cell types via lineage tracing, which is considered the gold-standard to assay stem cell function in vivo, has permitted the identification of several gastric stem cell markers, which will be the focus of the following sections.

The search for gastric stem cell markers revealed that there were several populations of active stem cells and reserve stem cells per gland, often with common genes marking populations in both the antrum and corpus. Homeostasis of the corpal epithelium is maintained by stem cells located in the highly proliferative isthmus region marked by *Mist1*, *Runx1*, *Sox2* and *Troy*, although lineage tracing has been observed occasionally from Troy+ cells located in the base [32]. However, there is a population of reserve stem cells located in the base of the corpus gland which can give rise to whole corpus glands in response to injury which is marked by several genes including *Mist1*, *Lgr5*, *Runx1*, *Sox2* and *Troy* [189,190,191,192]. Four stem cell markers have been identified in the antrum; *Lgr5*, *Sox2*, *Lrig1* and *CCKBR* [103,119,189,193], which all contribute to homeostasis of this tissue. Below is a description of each of the gastric stem cell markers identified to date. 

### 3.3. Lgr5

In 2010 Barker et al identified a small population of rapidly cycling cells that expressed the Wnt target gene *Lgr5* at the base of antral glands [103]. To determine if this population of cells were stem cells they generated *Lgr5-EGFP-IRES-CreERT2/Rosa26lacZ* mice and performed lineage tracing in vivo. Lgr5+ cells were indeed able to give rise to all the differentiated cells of the antral epithelium, demonstrating they are long-lived multipotent stem cells of the gastric epithelium [88]. Lgr5+ gastric stem cells share similar proliferation kinetics with their intestinal counterparts, demonstrating a drift towards clonality and regular mitotic activity [194]. Isolated Lgr5+ stem cells form long-lived gastric organoids that closely resembled the antral epithelium, demonstrating this population of cells has potent stem cell qualities [88]. These gastric organoid cultures require Wnt3a and R-Spo in the culture medium highlighting that Wnt is needed to maintain the gastric epithelium [103,195]. 

Similar expression patterns were seen in adult human antrum via in-situ hybridization, suggesting *LGR5* also marks gastric stem cells in humans [103]. However, transgenic silencing of the *Lgr5-EGFP-IRES-CreERT2* locus resulted in variegated expression of this locus and thus limited the investigation into the full extent of *Lgr5* as a stem cell marker. To overcome this, Leushacke et al. recently developed a non-variegated mouse model, *Lgr5-2A-CreERT2*, to allow further investigation of Lgr5. They were now able to detect that *Lgr5* was also expressed in a sub-population of chief cells at the base of corpal glands [191]. Lineage tracing showed that although this population of cells do not give rise to fully traced glands during homeostasis, they were activated in response to damage, after which they could give rise to whole corpus glands [191]. This highlights *Lgr5*-expressing cells as reserve stem cells in the corpus but as more active stem cells in the antrum where they contribute to daily tissue homeostasis. Ablation of Lgr5+ cells in the corpus resulted in compromised tissue architecture and a substantial reduction in the capacity to form corpal stomach organoids [191]. Although these data indicate the importance of *Lgr5* in regulating homeostasis in the corpus they also suggest that reserve stem cells are functioning to maintain corpus function when *Lgr5* is deleted. This is consistent with the present model of several populations of stem cells co-operating during homeostasis and damage in the gastric epithelium.

### 3.4. RunX1

The transcription factor Runx1 is required for the generation of hematopoietic stem cells and endothelial cells [196], and is expressed in several epithelial tissues including breast [197] and skin [198]. Investigations into the expression of Runx1 in the gastric epithelium via in-situ hybridization (ISH) and immunofluorescence analyses on tissues from C57BL/6 mice revealed that it marks a population of highly proliferative cells located in the isthmus [199]. An element of the *Runx1* promoter called *eR1*, was identified as an enhancer of *Runx1* in hematopoietic stem cells [200,201]. Lineage tracing experiments on the gastric tissue from *eR1-CreERT2;Rosa-LSL-tdTomato* and *eR1-CreERT2;Rosa-LSL-EYFP* mice revealed that eR1 marked a population of stem cells in the isthmus of the corpus and the antrum [185]. These eR1+ stem cells continuously gave rise to differentiated cells to maintain the gastric unit as well as generated gastric organoid cultures in vitro when co-cultured with Wnt3a, EGF, Noggin and R-Spo. Rarer Runx1+ chief cells at the base of the corpus can also function as reserve stem cells that function, along with other populations of gastric stem cells, during repair [185]. 

### 3.5. Sox2

The transcription factor *Sox2* is expressed in a variety of adult self-renewing epithelia including oesophageal, lung, intestinal and stomach [189]. *Sox2-CreERT2;Rosa26-LSL-EYFP* mice identified that *Sox2* is expressed in the corpus and antrum of the gastric epithelium, with some clusters of EYFP expression demonstrating that some rare Sox2+ cells had expanded over the 4-day pulse period [175]. 15–22 months after the treatment fully traced glands in both the corpus and antrum were observed, suggesting that Sox2+ cells have the ability for self-renewal and can give rise to the mature cell types of the glandular stomach. The fully traced corpus glands were seen less frequently than the antrum glands which is consistent with the reported slower turnover rate of corpus cells (3–194 days) compared with antral cells (1–60 days). 

Co-labelling experiments with antibodies that recognized known differentiated cell types of the stomach showed that Sox2+ cells were negative for all differentiation markers in the antrum and corpus and therefore are uncommitted stem cells. However, EYFP+ glands originating from lineage-traced Sox2+ cells 6 months after the treatment showed co-staining of EYFP+ cells with markers of enteroendocrine cells, mucus cells, parietal cells and chief cells. It can therefore be concluded that Sox2+ cells are multipotent stem cells in both the antrum and the corpus and the observation that labelled glands were still detectable up to 460 days after the treatment highlight Sox2+ cells self-renewing ability.

Since Barker et al demonstrated that Lgr5 cells are multipotent stem cells in the antrum, it was important to assess if there was any overlap in the expression patterns of *Sox2* and *Lgr5*. IHC for GFP and Sox2 on sections of antrum tissue from *Lgr5-GFP-IRES-CreERT2* mice showed no overlap in the expression of *Lgr5* and *Sox2*, suggesting these genes mark district types of stem cells in the antrum. In the corpus, *Troy* expression was increased, whilst expression of *Sox2* remained unchanged in Lgr5 depleted corpus, suggesting these two populations may contribute to Lgr5-independent renewal [191].

### 3.6. Lrig1

Leucine-rich repeats and immunoglobulin-like domains 1 (Lrig1), an ErbB negative regulator, marks a population of multipotent cells in the corpus and antrum. Upon injury, these cells can proliferate to replace damaged gastric glands. Lineage tracing from *Lrig1-CreERT2;R26RLacZ* mice identified that Lrig1 marks a quiescent, long-lived intestinal stem cell population unique from Lgr5 [119]. Also, Lrig1 marks short- (24 h) and long-term (2 months) cells in the gastric epithelium. Further studies utilized an *Lrig1-CreERT2;R26R-YFP* mouse to demonstrate that Lrig1+ cells were located in the isthmus of the corpus, and towards the bottom, but not the base, of the antrum [202]. *Lrig1* is co-expressed with a subset of proliferating cells in both the corpus and the antrum, and a subset of parietal cells in the corpus [188]. Lrig1+ cells could give rise to long term (1-year post recombination) gastric lineage epithelial cells in both the corpus and the antrum [188], illustrating it is a marker of stem cells during homeostasis and injury repair in the gastric epithelium. 

Lrig1+ cells can also contribute to the recovery of gastric glands in the corpus after acute oxyntic injury induced by DMP-777, which is a parietal cell-specific protonophore. DMP-777 treatment of *Lrig1-CreERT2;R26R-YFP* mice identified that Lrig1+ cells gave rise to parietal cells (H/K-ATPase), neck cells (GS-II lectin), proliferating cells, and metaplastic cells (CD44v9) following tissue repair [188]. However, *Lrig1* knockout mice treated for three days with DMP-777, were able to recover from acute gastric mucosal injury, suggesting that Lrig1 protein is not required for regeneration of gastric mucosa after oxyntic atrophy and lineage differentiation [188]. 

### 3.7. Troy

Tumour necrosis factor receptor superfamily, member 19 (TROY), is a member of the TNF-receptor superfamily. In the stomach, *Troy* was originally reported to label cells at the base of corpus glands and pulse labelling from *Troy-eGFP-IRES-CreERT2* mice demonstrated that Troy was expressed in a small population of fully differentiated chief cells [192]. Although Troy+ chief cells do not proliferate, or if so very rarely, they can form organoids, suggesting they have stem cell properties in specific situations [178]. Indeed, lineage tracing in *Troy-eGFP-IRES-CreERT2* mice showed that fully traced glands did originate from Troy+ chief cells but at a very slow rate, often taking up to 3 months or onward after recombination [178]. These data suggest Troy+ chief cells act as reserve stem cells in the corpus. In support of this, treatment of *Troy-eGFP-IRES-CreERT2* mice with 5-fluorouracil (5-FU) to selectively kill proliferative cells in the isthmus, greatly enhanced the rate and number of lineage tracing events originating from Troy+ chief cells in the corpus base [178]. Whole-labelled glands have also been reported to originate from a rare population of Troy+ cells in the isthmus of the corpus [32], suggesting there are two populations of Troy+ cells in the corpus; one in the isthmus which is able to lineage trace during normal homeostasis [174], and another in the base of the glands which functions predominantly as a population of reserve stem cells during healing [203].

### 3.8. Mist1

Mist1 is a basic helix-loop-helix transcription factor. Using *Mist1-CreERT2-R26-mTmG* mice it was observed that Mist1 is expressed predominantly in chief cells of the lower two thirds of the corpus, with a rare population also observed in the isthmus [174]. Lineage tracing revealed bi-directional expansion from a single Mist1+ cells in the isthmus which gave rise to long term, fully traced glands containing all the differentiated cell types of the corpus [174], illustrating Mist1 marks stem cells located in the isthmus. *Mist1-CreERT2;R26-Confetti* mice revealed that Mist1+ isthmus cells gave rise to blocks of colour expansion, whilst recombination in chief cells did not, thus demonstrating that Mist1+ cells located in the isthmus are the predominant stem cell in the corpus rather than chief cells [174]. In support of this, isthmal Mist1+ cells were able to form gastric organoids while Mist1+ chief cells remained as single cells and did not form organoids. To determine if Mist1+ cells were giving rise to Lgr5+ cells when cultured for organoid growth, Lgr5 cells were first ablated in *Mist1-CreERT2;Lgr5-DTR;R26-TdTomato* mice, however this did not prevent robust organoid culture from isthmus Mist1+ cells highlighting Mist1+ cells are capable of self-renewal in the absence of Lgr5+ cells.

### 3.9. CCK2R

Cholecystokinin B receptor (CCK2R) is the receptor for gastrin and pro-gastrin which are expressed by G-cells in the antral stomach. *CCK2R-CreERT-BAC* mice revealed that CCK2R expressing cells were located at +3 to +7 position above the base of the antral gastric units [193]. Lineage tracing from *CCK2R-CreERT2-BAC;Rosa26TdTomato* mice revealed that CCK2R+ cells were able to give rise to full antral gastric units containing all differentiated cells, and were thus markers of antral stem cells [179]. In support of this, FACS sorted CCK2R+ cells could grow gastric organoids, whilst CCK2R negative cells failed to do so. CCK2R+ cells were negative (or very low) for *Lgr5* expression, suggesting they are a distinct population, and are also located slightly above the Lgr5+ population and were more proliferative [179]. However, treatment of Lgr5neg or low/CCK2R+ gastric cells with progastrin resulted in rapid interconversion of these cells into Lgr5high cells, thus expanding the pool of active stem cells in the antrum.

### 3.10. How Wnt Signalling Regulates Gastric Stem Cells

Wnt signalling is well characterised as a critical signalling pathway regulating homeostasis and stem cell function in the intestine (Table 1). Among the first reports to demonstrate this were loss of stem cells and proliferative cells when the Wnt antagonist Dickkopf1 (Dkk1) was overexpressed in the intestine [89,90], and the failure of crypts to form in Tcf4−/− mice [16]. However, there is growing evidence that Wnt is also important in the gastric epithelium. Gastric organoid cultures require Wnt3a and R-Spo in the culture media [103,195] demonstrating that gastric stem cells require Wnt. Indeed, the first stem cell marker identified in the intestine, Lgr5, is also a marker of gastric stem cells, and has been identified as a Wnt target gene, and a co-receptor involved in Wnt signalling whereby it promotes Wnt signalling by associating with R-Spo to titrate RNF43/ZNRF3 and thus reduce the turnover of Fzd receptors on the cell surface [103,148]. These data highlight that Wnt signalling is active in the gastric epithelium, with further investigation identifying that Wnt signalling is higher in the antrum than the corpus [18], although Troy+ cells in the base of the corpus also express Wnt target genes and stem cell signature genes [192]. 

There is also evidence of co-operation between Wnt1 and the gastric stem cell marker, and Wnt target gene CD44. Overexpression of *Wnt1* dramatically improves the spheroid formation of gastric cancer cell lines as well as enriches the side population cells with stem-like properties. Of interest, *CD44* and *β-catenin* are upregulated in cells overexpressing *Wnt1* [204]. Therefore, a subpopulation of gastric stem cells may be present whereby *Wnt1* overexpression increases the relative prevalence of these cells. *CD44* was also upregulated during reprogramming of chief cells, induced by deletion of *Mist1* as they underwent SPEM, suggesting Wnt may be regulating this process [205]. However, *CD44* is also regulated by other pathways in the gastric epithelium, including p-ErK–CD44–pStat3 signalling that has been demonstrated to regulate SPEM [191].

Detailed analysis of Wnt signalling in the epithelium of the antrum using in situ hybridization (ISH) showed that several Wnt ligands were expressed (Wnt2b, 3a, 4, 5a, 9a, 9b and 11), and all 10 Fzd receptors [29]. The expression patterns of some Fzd receptors was focused in specific areas of the gastric units, for example Fzd10 was expressed predominantly in the pit region, Fzd6 predominantly in the base, Fzd5 expression was markedly decreased in the gland base, whilst Fzd6 and Fzd7 expression was mainly confined to the bottom two thirds of the gastric unit [192]. These specific expression patterns suggest a distinct role for Wnt signalling in each area of the gastric unit, which has yet to be fully elucidated and will no doubt become clearer in future studies.

Using an *Axin2-CreERT2* mouse and ISH, the location of canonical Wnt responsive cells in the antrum has recently been revealed to be constrained to the gland neck and base [192]. Axin2+ cells were undifferentiated, and lineage tracing revealed they could give rise to full glands containing differentiated cells, and thus marked a population of stem cells. Interestingly, only a proportion of Axin2+ cells co-express Lgr5, and Axin2+ cells were still able to lineage trace in *Lgr5DTR* mice in which Lgr5+ cells had been depleted, indicating two distinct populations of Wnt sensitive stem cells located in the antral gland base [192]. Axin2+/Lgr5− cell activity was regulated by R-Spo3 secreted by the underlying myofibroblasts in vivo, however, this was not observed for less proliferative Lgr5+ cells that were also insensitive to R-Spo1 or R-Spo3 treatment in vivo [192].

Despite evidence that Wnt signalling is active in the gastric epithelium, it has only recently been discovered which Fzd receptor transmits these signals. In addition to its expression in the intestinal epithelium, [14], we have recently shown that *Fzd7* is also expressed in the antrum of the gastric epithelium and deletion of this receptor in vivo is deleterious and triggers rapid repopulation of the epithelium [18]. This was the first observation that the gastric epithelium has the capacity to repopulate following genetic insult, in this case deletion of *Fzd7*, and also the first demonstration that Wnt signalling is critical in the regulation of the antral epithelium [18]. Fzd7 can transmit signalling via the canonical and non-canonical pathways. However, in the gastric epithelium Fzd7 is most likely to be transmitting signals via the canonical pathways since gastric organoid death upon *Fzd7* deletion could be rescued by activating the canonical Wnt pathway at the level of GSK3β [18]. The expression of several canonical Wnt target genes are reduced following *Fzd7* deletion, however, this does not preclude that non-canonical signalling may still be involved in regulating gastric stem cells, and homeostasis. Indeed, Wnt5a regulates gastric tumour progression [206], whilst Wnt5a-Ror2 regulates mesenchymal stem cells to promote the growth of gastric cancer cells [207], suggesting this pathway may also regulate gastric stem cells. In the intestine, Fzd7 is required for the function of Lgr5+ cells [14]. However, although Fzd7 clearly regulates gastric homeostasis, the exact population of stem cells that require Fzd7 has yet to be identified. 

### 3.11. The Oncogenic Role of Wnt Signalling in Gastric Cancer

Gastric cancer is a common malignancy with approximately 1,000,000 new cases diagnosed annually and is the third most common form of cancer-related death world-wide [208]. The largest proportion of cases are reported from East Asia, South America and Eastern Europe [209]. Gastric cancer is divided histopathologically into two classes: diffuse-type and intestinal-type [210]. However, these broad classifications are dwarfed by the complex pathogenic, environmental and genomic milieu that ultimately shape the characteristics of any given gastric lesion. In terms of medical interventions, surgical resection is the first option, but is only effective if gastric tumours are detected early, which unfortunately is seldom. Encouragingly, various therapies targeting essential signalling pathways have provided some therapeutic benefit to patients, but are often fraught with drug-acquired resistance and tumour relapse, highlighting the need to identify other druggable rate-limiting pathways utilized by gastric cancer cells. Wnt signalling is deregulated in gastric cancer at several points along the pathway including the ligand/receptor interface, regulatory proteins and transcriptional machinery. Moreover, the link between aberrant Wnt signalling and cancer is well established in many solid tumours including breast, liver and the colon. However, its functional relevance in gastric cancer is less well characterized. In the following section, we review the extent of Wnt signal deregulation in the stomach and the cellular and molecular consequences. 

Chronic infection with the gram-negative bacteria *Helicobacter pylori*, which has evolved the capacity to survive in arguably the most hostile environment of the human body, the stomach, is a major risk factor for the development of gastric cancer [211]. Following the successful colonization of the stomach lumen, *H. pylori* migrates proximally to the stomach epithelium where it delivers bacterial virulence factors that modulate epithelial biology and inflammatory responses for its own benefit. Of the virulence factors produced by *H. pylori*, cytotoxin-associated gene product (CagA) and its associated type IV secretion system (T4SS) have been shown to activate Wnt signalling and promote gastric tumourigenesis and progression [211,212]. Thomas Meyer’s group demonstrate that virulent strains of *H. pylori* directly inject CagA into gastric epithelial cells, including Lgr5+ gastric stem cells [213]. Indeed, the number Lgr5+ gastric cells is significantly increased following infection with *H. pylori*, which was associated with an increase in Wnt signalling activity and enhanced clonogenic potential of ex vivo cultures [213]. Further refinement of previous host-pathogen interaction models reveal a novel population of Wnt responsive *Axin2^+^* gastric stem cells significantly expand following *H. pylori* infection, which is associated with an increase in R-Spo3 signalling from subepithelial myofibroblasts [29]. Indeed, both R-SPO and LGR5 (the receptor for R-SPO) expression are positively correlated with poor patient survival and outcome [214,215]. Gastric epithelial cells infected with *H. pylori* induce the expression and subsequent activation of other Wnt receptor components such as Fzd [216] and Lrp [217], which propagate Wnt signalling and cell transformation. Collectively, these data provide a rational therapeutic foundation to target Wnt signalling at the level of the receptor complex in *H. pylori* infected gastric cancer cells. 

Independent of *H. pylori*-mediated activation of Wnt signalling, mutation and epigenetic modification at multiple tiers of the Wnt pathway feature in subsets of gastric cancer patients [218]. For instance, LOF mutations to the E3 ubiquitin-protein ligase *RNF43* are reported in 54% and 8% of MSI and microsattelite stable (MSS) gastric tumours respectively [219]. The functional consequence of *RNF43* mutation allows for FZD receptor stabilization, hyper-sensitivity to available Wnt ligands and subsequent Wnt activation [151]. Diagnostically, *RNF43* mutations occur in early-stage gastric lesions [220], which could be used to stratify patients who may benefit from compounds that block the secretion of Wnt ligands, which have substantial efficacy in other ‘Wnt-addicted’ *RNF43* mutant cancers [221,222]. Another common mechanism to de-regulate Wnt signalling is via forced stabilization of β-catenin, which facilitates robust transcription of Wnt target genes [223], many of which regulate multiple hallmarks of cancer [224]. Stabilisation and accumulation of β-catenin can occur via truncating *APC* [219,225], which is a core component of the β-catenin destruction complex. Of note, allelic imbalance of *APC* can be detected in the blood and saliva of patients with gastric adenocarcinoma and proximal polyposis of the stomach (GAPPS) [226], which place APC as an excellent prognostic marker. Gastric tumours expressing nuclear β-catenin often harbor gain-of-function mutations in *CTNNB1* (encoding β-catenin) [227,228], which permit β-catenin to escape proteosomal degradation and initiate transcription of target genes involved in cell proliferation and growth [229,230]. As such, targeted inhibition of β-catenin in gastric cancer cells is sufficient to inhibit Wnt signalling, cell proliferation and induce apoptosis via suppression of Survivin [231]. 

Other regulators of the Wnt pathway such as Dickkopf (*DKK*) and secreted Frizzled-related protein (*sFRP*) are epigenetically silenced via promoter hypermethylation in gastric cancer patients, which permit excessive Wnt activation. Importantly, treatment with de-methylating agents or exogenous expression of *sFRP* or *Dkk* is sufficient to arrest gastric cancer growth in vitro and in tumour xenografts [232,233,234]. Interestingly, potent anti-tumour effects of functional Dkk and/or sFRP are observed in gastric cancer cells with and without truncating mutations to *APC*, which provide compelling evidence that Wnt signalling can be further modulated in *APC* mutant gastric cancer cells. 

Despite the low incidence of genetic lesions to Wnts and Fzd receptors reported in gastric cancer, aberrant expression of Wnts and Fzd receptors is often observed in gastric tumours [25,218]. The prototypical ‘non-canonical’ Wnt, Wnt5a, which is synonymous with Wnt/β-catenin inhibition and promotion of cell motility and migration [235], is overexpressed in gastric cancer and correlates with poor outcome [236]. Hayakawa and colleagues elegantly demonstrate Wnt5a, which is secreted from a perivascular niche, permit Apc/Kras mutant Mist1+ cells in the corpal stomach to circumvent anoikis, providing a permissive space for anchorage-independent growth. In support, Wnt5a-deficient bone marrow chimeras or treatment with polyclonal anti-Wnt5a antibody is sufficient to reduce gastric tumourigenesis [32,178]. Like its binding counterpart, Fzd receptors show de-regulated expression in gastric cancer patients [237], which is likely attributed to previously discussed Wnt-activating mutations. Of the ten mammalian FZDs, *FZD7* is overexpressed in gastric cancer at various stages of disease progression [237,238,239], which reflects its promiscuous capability to transduce Wnt signalling via all major signalling arms [14,52]. Together, these data demonstrate gastric cancer cells commonly display aberrant levels of Wnt signalling, which is rate-limiting for efficient tumour growth and thus, warrants further investigation into safely targeting Wnt signalling in gastric cancer. 

Functional proof that deregulated Wnt signalling can trigger gastric tumourigenesis was first demonstrated following genetic deletion of *Gsk3-β* in the mouse gastric epithelium, which developed intestinal type adenomas [103,240]. The *Apc* floxed allele has also been used to illustrate that gastric tumourigenesis can be triggered upon deregulation of Wnt signalling, however, *Mist1Cre;Apc^fl/fl^* mice only develop tumours in the antrum [241], despite robust recombination and deregulation of Wnt signalling in both the corpus and antrum [32], highlighting that the antrum is more sensitive to Wnt signalling. *Sox2Cre;Apc^fl/fl^* mice develop gastric tumours in the corpus and antrum, suggesting that each stem cell population in the gastric epithelium has a distinct response to this level of deregulated Wnt signalling, as gastric tumourigenesis was not observed in the corpus of *Mist1Cre;Apc^fl/fl^* mice [25]. Gastric tumours did develop in the corpus of *Mist1Cre;Apc^fl/fl^;Kras^G12D^* mice and *TroyCre;Apc^fl/fl^;Kras^G12D^* illustrating a co-operation between Wnt and Kras in gastric tumourigenesis, and that stem cells are the cell of origin in the gastric epithelium [32]. Indeed, *Lrig1Cre;Apc^fl/+^* mice also developed gastric tumours in the antrum, as well as the intestine [242].

## 4. Summary

There is currently a vast body of data highlighting the critical role of Wnt signalling in regulating many functions of stem cells in the gastric and intestinal epithelium, including survival, proliferation and clonality. These are also cell functions which are hijacked via aberrant Wnt signalling during transformation by oncogenes or loss of tumour suppressors, which has also been well documented in gastric and intestinal cancer, highlighting the importance for strict regulation of the Wnt pathway to prevent pathologies. It also highlights the great potential to harness these regulatory properties to enable scientists and clinicians to manipulate cell function to their own specific needs. For example, a transient increase in proliferation could greatly aid regenerative medicine [243], whilst well defined Wnt activity is critical for the culture of organoids and tumour organoids from several tissues including the stomach and intestine [244]. To fully understand how Wnt signalling regulates stem cells, and thus triggers pathologies, current and future projects are trying to elucidate the complex interactions between the different stem cell populations and how signalling pathways interact with each other to regulate these functions [245]. A good barometer of the journey left to travel on this research path is the relatively few specific roles that can be assigned to a single Wnt ligand or receptor which will eventually illuminate the full extent of how this pathway network regulates this import population of cells.

## Figures and Tables

**Table 1:** The effects of Wnt ligands on gastrointestinal stem cell populations during homeostasis and cancer.

Gene	Expression	Homeostasis	Cancer	Reference
*WNT1*	Gastric epithelium	Gastric stem cell maintenance. Regulates Oct4.	Overexpressed in GC.	[25]
*WNT2*	Gastric epithelium	Unknown.	Overexpressed in GC and CRC. Promotes cell reprogramming.	[26]
*WNT2B*	Gastric gland base, intestinal stroma	Unknown, Intestinal stem cell maintenance.	Over expressed in CRC and GC.	[27,28,29]
*WNT3A*	Paneth cell/crypt	Intestinal stem cell maintenance.	Over expressed in CRC and GC.	[28,30]
*WNT4*	Gastric isthmus, intestinal mesenchyme/epithelium	Unknown, intestinal crypt maintenance/regeneration.	Increased expression in GC.	[27,29]
*WNT5A*	Corpus stroma, intestinal villi, colonic epithelium	Gastric stem cell niche, colonic regeneration.	Promotes invasion and prevents anoikis in GC.	[31,32,33]
*WNT6*	Paneth cell/crypt, gastric gland	Intestinal stem cell maintenance, supports gastric niche.	Over expressed in GC.	[29,33,34]
*WNT9A*	Gastric isthmus and base	Unknown.	Over expressed in CRC and GC.	[29,35]
*WNT9B*	Paneth cell/crypt gastric isthmus	Intestinal stem cell maintenance, unknown.	Unknown.	[29,33]
*WNT10B*	Gastric epithelium	Unknown.	Required for proliferation and migration in GC.	[29,36]
*WNT11*	Gastric isthmus, surface pit and neck	Unknown.	Increased expression in CRC.	[29,37]
*WNT16B*	Gastric epithelium	Unknown.	Overexpressed in GC.	[38]
